# Hyperinsulinaemic hypoglycaemia: A rare association of vanishing white matter disease

**DOI:** 10.1002/jmd2.12081

**Published:** 2019-11-12

**Authors:** Carolyn Bursle, Eppie M. Yiu, Alison Yeung, Jeremy L. Freeman, Chloe Stutterd, Richard J. Leventer, Adeline Vanderver, Joy Yaplito‐Lee

**Affiliations:** ^1^ Department of Metabolic Medicine Royal Children's Hospital Melbourne Australia; ^2^ Department of Neurology Royal Children's Hospital Melbourne Australia; ^3^ Murdoch Children's Research Institute Melbourne Australia; ^4^ Department of Paediatrics University of Melbourne Melbourne Australia; ^5^ Victorian Clinical Genetics Service Melbourne Australia; ^6^ Neurology Department Children's Hospital of Philadelphia Philadelphia Pennsylvania

**Keywords:** EIF2B, hyperinsulinism, hypoglycemia, leukodystrophy, leukoencephalopathy, vanishing white matter

## Abstract

We report two unrelated patients with infantile onset leukoencephalopathy with vanishing white matter (VWM) and hyperinsulinaemic hypoglycaemia. To our knowledge, this association has not been described previously. Both patients had compound heterozygous pathogenic variants in *EIF2B4* detected on exome sequencing and absence of other variants which might explain the hyperinsulinism. Hypoglycaemia became apparent at 6 and 8 months, respectively, although in one patient, transient neonatal hypoglycaemia was also documented. One patient responded to diazoxide and the other was managed with continuous nasogastric feeding. We hypothesise that the pathophysiology of hyperinsulinism in VWM may involve dysregulation of transcription of genes related to insulin secretion.

## 1 INTRODUCTION

Vanishing white matter (VWM, OMIM 603896) is also known as childhood ataxia with central nervous system hypomyelination (CACH), and is one of the most common leukodystrophies.^1^ VWM encompasses a spectrum of clinical disease, with the most severe form having early infantile or even prenatal onset, and the least severe form being characterised by adult onset and slow progression, with ovarian dysgenesis.^2^ Age of onset is most frequently in early childhood, with prominent cerebellar ataxia and spasticity. Cognitive deterioration, epilepsy and optic atrophy can occur as the disease progresses.^3^, ^4^, ^5^ Rapid neurological deterioration can be triggered by stresses such as febrile infection, minor head trauma, and even acute fright.^3^, ^5^, ^6^, ^7^, ^8^

Diagnosis of VWM is based on characteristic MRI abnormalities.^2^ Pathogenic variants in one of five genes (*EIF2B1, EIF2B2, EIF2B3, EIF2B4, EIF2B5*) encoding the five subunits of the eukaryotic translation initiation factor 2B (eIF2B) are found in ~90% of individuals with VWM.^9^ Characteristic MRI findings include diffuse, symmetric cerebral white matter involvement, with low T1, proton density, and FLAIR images and high signal on T2 images. There is relative sparing of U‐fibres, internal capsules, anterior commissure and the outer rim of the corpus callosum.^2^, ^9^ These abnormalities can be present in presymptomatic individuals.^5^, ^7^, ^10^, ^11^ Over time, there is progressive rarefaction and cystic degeneration of the affected white matter, which is eventually replaced by CSF‐like fluid.^5^, ^7^ The cerebellar white matter and brainstem may also be involved, but with atrophy rather than cystic degeneration.^5^, ^7^

## 2 METHOD

Patients 1 and 2 were assessed by the Royal Children's Hospital Metabolic and Neurology services. DNA was extracted from whole blood from both patients and exome sequencing performed using the Nextera Rapid Capture Exome kit (Illumina). Sequencing was performed on an *Illumina HiSeq4000*. Data was processed using Cpipe.^12^ Curation of variants was phenotype‐driven with relevant gene lists used for variant prioritisation. Classification of variants was based on ACMG guidelines.^13^

## 3 PATIENT 1

This patient is the second child born to healthy nonconsanguineous parents. He was born at 36 weeks and 5 days by emergency caesarean section due to decreased fetal movements and amniotic fluid index. This occurred in the setting of gestational diabetes and decreasing maternal insulin requirements suggestive of placental insufficiency. Delivery was uncomplicated and Apgars were 9 at 1 and 5 minutes. Respiratory distress developed on day 1 with a small pneumothorax noted. Surfactant was given and CPAP was delivered for 2 days. Hypoglycaemia did not occur in the neonatal period.

The infant's early motor development was mildly delayed, unilateral sensorineural hearing loss was noted, and cows' milk protein allergy was diagnosed. At age 6 months, he presented with status epilepticus in the context of hypoglycaemia (1.8 mmol/L measured by dextrometer) although the subsequent formal glucose level was 2.9 mmol/L and the insulin level was 5mIU/L. This occurred on a background of 5 days of poor feeding, lethargy, developmental regression with hypotonia and focal seizures characterised by right sided facial and upper limb clonic jerking. He was commenced on maintenance levetiracetam. A further hypoglycaemic episode was recorded in hospital with a blood glucose level of 1.9 mmol/L, less than 2.5 hours after a nasogastric bolus feed. This occurred in the context of a mild bronchiolitic illness and single vomit several hours prior to the documented hypoglycaemia. Critical samples including insulin level, free fatty acids, and β hydroxybutyrate were not obtained at the time of hypoglycaemia, but urine organic acids and acylcarnitine profile were normal, without evidence of ketosis.

**Figure 1:**
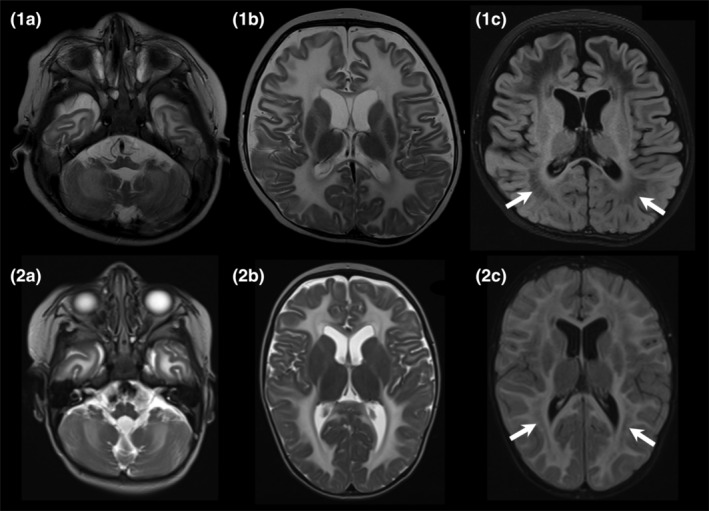
Top row is patient 1 (age 6 months) and bottom row is patient 2 (age 9 months). The images in the two left columns are axial T2‐weighted at 1.5 T showing a diffuse leukoencephalopathy with symmetric high signal throughout the cerebral and cerebellar white matter. There is relative sparing of the internal capsules. The images in the right column are FLAIR sequences showing rarefaction of the white matter (arrows)

MRI brain performed at 6 months of age showed marked T2 hyperintensity in the supra‐ and infra‐tentorial white matter with extensive diffusion restriction. (Figure 1) The possibility of Aicardi‐Goutieres syndrome was raised due to the MRI appearance but CT brain did not show calcifications and CSF neopterin was not elevated. Extensive metabolic workup including paired blood and CSF samples was unrevealing. Chromosome microarray was normal.

Hypoglycaemia was an ongoing but intermittent problem, with fasting times of less than 4 hours. Seizures continued to occur infrequently on levetiracetam monotherapy. There was slow developmental progress, including improved head control and the ability to sit briefly unsupported by age 9 months. He had a gastrointestinal illness aged 10 months without developmental regression, but with recurrent hypoglycaemia which persisted after resolution of the illness. Continuous nasogastric feeding was instituted, and attempts to reinstate a bolus regimen were unsuccessful due to recurrence of hypoglycaemia. At 12 months, he was admitted for investigation and management of the ongoing hypoglycaemic episodes. Hyperinsulinism was noted, with several instances of inadequate suppression of insulin in the context of hypoglycaemia. In one episode, glucose was 2.3 mmol/L and insulin 5 mIU/L. Cortisol and growth hormone levels were normal. Hypoglycaemia occurred despite glucose delivery rate (GDR) of 9 mg/kg/min via continuous nasogastric feed. Hypoglycaemia resolved after diazoxide was commenced at 8 mg/kg/d and weaned to 5 mg/kg/d later.

Unfortunately, during this admission he developed a Parainfluenza 3 related lower respiratory tract infection. Rapid neurological deterioration ensued, necessitating intubation and ventilation. A repeat MRI showed progression of the white matter abnormality, with rarefaction of affected white matter and atrophy of the brainstem and cerebellum, suggesting a diagnosis of VWM. There was abnormal signal in the basal ganglia and cerebellum, and restricted diffusion of the cerebellum and subcortical U fibres. After 1.5 weeks without improvement, and a failed extubation attempt, he became bradypnoeic with irregular respiration. Death occurred soon after extubation. Muscle, liver and skin biopsies were taken for respiratory chain enzymology, and were stored pending genetic studies.

### 3.1 Exome sequencing results

Exome sequencing detected compound heterozygous missense variants in the *EIF2B4* gene: a paternally inherited c.725C>T (p.Pro242Leu) variant and a maternally inherited c.1301T>C (p.Leu434Pro) variant. The c.725C>T variant results in a moderate amino acid change at a highly conserved residue within the IF‐2B domain. It has been previously described in four homozygous patients with EIF2B4‐related leukodystrophy.^14^ The c. 1301 T>C variant results in a moderate amino acid change at a highly conserved residue located within the IF‐2B domain and is absent from population databases (gnomAD, dbSNP, 1000G). In silico predictions for this variant are consistently pathogenic (Polyphen, SIFT, CADD, Mutation Taster). No pathogenic changes were detected to explain the hyperinsulinism, with the following genes being analysed: ABCC8, AKT2, GCK, GLUD1, HADH, HNF1A, HNF4A, INS, INSR, KCNJ11, MPI, PDX1, SLC16A1, USHIC, UCP2. Adequate coverage of these genes was observed, with a mean of 91% of bases sequenced at greater than ×20.

## 4 PATIENT 2

Patient 2 was born at term, to healthy nonconsanguineous parents whose only previous pregnancy had ended in unexplained stillbirth. He had apnoeas and hypoglycaemia on day 3 of life and was given antibiotic cover for suspected sepsis. Hypoglycaemia persisted, with intravenous dextrose being given for 11 days, and the cause being attributed to a difficult delivery and possibly infection. Urine metabolic screen and organic acids were normal and plasma amino acids were nondiagnostic. Newborn screening was normal. A microarray was performed because of subtle dysmorphism and the result was normal. By 5 months of age, there were concerns with recurrent vomiting, slow development and lethargy. Mild developmental regression was noticed over the following months. At 8 months of age, he was admitted to the hospital due to failure to thrive and accelerated regression of motor skills over the preceding 3 weeks. He lost the ability to roll, a motor skill previously gained at 5 months. He could sit briefly unassisted in a tripod position. He had axial and appendicular hypotonia with poor head control.

Recurrent hypoglycaemia was noted in hospital, despite 3 hourly bolus feeds with GDR of 6.2 mg/kg/min. On one occasion, glucose was 1.9 mmol/L and insulin was 2.1 mU/L. Guthrie card acylcarnitines were normal several hours later. He was asymptomatic during the hypoglycaemic episodes. There were difficulties in obtaining a full hypoglycaemia screen, and additional insulin levels were not obtained at the time of hypoglycaemia. However, ketones were low when tested during hypoglycaemic episodes consistent with hyperinsulinism. Continuous nasogastric feeds were instituted with a GDR of ~9 mg/kg/min, with resolution of the hypoglycaemia.

Extensive biochemical investigations on blood and CSF were unrevealing. A trial of thiamine, riboflavin and CoQ10 was commenced, but was limited by vomiting. Brain MRI at 9 months was suggestive of a leukodystrophy, with confluent cerebral and cerebellar white matter signal changes, with lesser degree of involvement of the corpus callosum, internal capsule and brainstem, with patchy globus pallidi signal change. The pattern was suggestive of myelin destruction. (Figure 1).

Neurological deterioration occurred rapidly, with seizures, increased peripheral tone and apnoeas. He died at age 10 months.

### 4.1 Exome sequencing results

Exome sequencing detected compound heterozygous missense variants in the *EIF2B4* gene. A maternally inherited variant, c.725C>T (p.Pro242Leu) and a paternally inherited variant, c.628G>T (p.Gly210Cys) were identified. The first variant was the same recurrently reported variant found in Patient 1. The second c.628G>T variant results in a major amino acid change at a highly conserved residue and is absent from population databases (gnomAD, dbSNP, 1000G). In silico predictions consistently report this variant to be pathogenic (Polyphen, SIFT, CADD, Mutation Taster).

## 5 DISCUSSION

Disease mechanisms in VWM are incompletely understood. eIF2B is an evolutionarily conserved protein complex involved in translation initiation.^15^, ^16^, ^17^ The eIF2B‐catalysed step of GDP/GTP exchange is rate‐limiting in translation initiation, regulating the rate of global protein synthesis.^15^ eIF2B activity is regulated by numerous factors, including phosphorylation of the eIF2α subunit, which inhibits mRNA translation by acting as a competitive inhibitor of eIF2B.^18^ During cellular stress, a mechanism known as the unfolded protein response (UPR) is activated to inhibit protein synthesis and promote the degradation of unfolded or denatured proteins.^19^, ^20^, ^21^ Cellular stresses lead to eIF2α phosphorylation, inhibition of eIF2B and translation inhibition.^22^, ^23^, ^24^ As neurological deterioration is often triggered by stress in VWM patients, it has been hypothesised that the UPR drives the pathophysiology.^2^ While decreased eIF2B activity does not appear to affect the global rate of protein synthesis or cellular survival and proliferation in VWM patients,^25^, ^26^, ^27^, ^28^ evidence of activation of the UPR has been found in the white matter of VWM patients in vivo, and in cells with VWM mutations in vitro.^27^, ^28^, ^29^, ^30^ In VWM patients, the UPR appears to be activated in white matter astrocytes and oligodendrocytes, while other tissues with a high rate of protein synthesis may not be affected.^27^, ^28^ Recently, it has been observed that VWM cells hyper‐repress translation during stress conditions, and there is disruption of a negative feedback loop which allows stress adaptation and translational recovery in healthy cells (Moon and Parker, 2018).^31^

Insulin secretion is complex, and synthesis is regulated at both the transcription and translation levels. The speed of protein translation in pancreatic β cells is increased in response to nutrients. This process is partly controlled by dephosphorylation of eIF2a.^32^ Insulin rapidly activates protein synthesis via mechanisms including activation of protein kinase B which phosphorylates and inactivates glycogen synthase kinase 3, which leads to the dephosphorylation of eIF2B, contributing to overall activation of protein synthesis.^33^

VWM can involve multiple systems, with ovarian dysgenesis being frequently reported, and in severe early onset cases, growth retardation, pancreatitis, renal hypoplasia, hepatosplenomegaly, and cataracts have been reported.^34^ The previously reported pancreatic involvement is intriguing in this context, but difficult to relate pathophysiologically to hyperinsulinism, as opposed to exocrine and endocrine insufficiency.

While it is unknown how eIF2b mutations might affect glycaemic control, intriguingly, the alpha subunit of eIF2B has been noted to associate with adrenergic receptors.^35^ In addition, endoplasmic reticulum stress signals, such as phosphorylation of PERK and eIF2a, have been induced by treatment of PC12 cells with noradrenaline.^36^ This raises the possibility of the acute fright and other stress responses seen in VWM being triggered by adrenaline or noradrenaline. None of the currently identified genes associated with hyperinsulinism encode proteins involved with adrenaline, noradrenaline or other counter‐regulatory hormones, however, a genetic cause is not identified in around 40% of cases of hyperinsulinism.^37^, ^38^

To our knowledge, hyperinsulinism has not previously been reported in VWM. A second pathology seems unlikely in nonconsanguineous families where exome testing did not reveal a cause of hyperinsulinism, however a relationship between hyperinsulinism and VWM is neither confirmed nor readily explained with current knowledge of the pathophysiology. We suggest the most likely pathogenic mechanism involves alteration of gene expression in genes involved in insulin regulation, potentially related to altered eIF2B activity in pancreatic beta cells.

## 6 CONCLUSION

We report two cases of hyperinsulinaemic hypoglycaemia in children with VWM disease associated with *EIF2B4* mutations, a phenomenon not previously reported. The mechanism by which *EIF2B* mutations may cause hyperinsulinism is not clear, but we hypothesise that it may be via the role of eIF2B's in translation initiation and regulation. It also remains to be seen whether there is a genotype/phenotype relationship, should the association be confirmed. In our patients, the hypoglycaemia was a significant management challenge, responding to continuous feeds at high GDRs or diazoxide. Clinicians should be alert to the possibility of hypoglycaemia complicating VWM disease, particularly in early onset cases, and consider the diagnosis of VWM in children with both a leukodystrophy and hypoglycaemia.

## COMPLIANCE WITH ETHICS GUIDELINES

The authors declare no potential conflict of interest.

## ETHICS APPROVAL

Specific ethics approval was not required.

## CONSENT

Informed consent was obtained from both families for the publication of this report.

## FUNDING SOURCE

Not applicable.

## ANIMAL RIGHTS

Not applicable.
